# A Decentralized Sensor Fusion Scheme for Multi Sensorial Fault Resilient Pose Estimation

**DOI:** 10.3390/s21248259

**Published:** 2021-12-10

**Authors:** Moumita Mukherjee, Avijit Banerjee, Andreas Papadimitriou, Sina Sharif Mansouri, George Nikolakopoulos

**Affiliations:** Robotics and AI Group, Department of Computer, Electrical and Space Engineering, Luleå University of Technology, SE-97187 Luleå, Sweden; avijit.banerjee@ltu.se (A.B.); andreas.papadimitriou@ltu.se (A.P.); sina.sharif.mansouri@ltu.se (S.S.M.); george.nikolakopoulos@ltu.se (G.N.)

**Keywords:** multi sensor fusion, decentralized fusion, linear minimum variance, maximum likelihood function, optimal information filter, fault resilient optimal information fusion

## Abstract

This article proposes a novel decentralized two-layered and multi-sensorial based fusion architecture for establishing a novel resilient pose estimation scheme. As it will be presented, the first layer of the fusion architecture considers a set of distributed nodes. All the possible combinations of pose information, appearing from different sensors, are integrated to acquire various possibilities of estimated pose obtained by involving multiple extended Kalman filters. Based on the estimated poses, obtained from the first layer, a Fault Resilient Optimal Information Fusion (FR-OIF) paradigm is introduced in the second layer to provide a trusted pose estimation. The second layer incorporates the output of each node (constructed in the first layer) in a weighted linear combination form, while explicitly accounting for the maximum likelihood fusion criterion. Moreover, in the case of inaccurate measurements, the proposed FR-OIF formulation enables a self resiliency by embedding a built-in fault isolation mechanism. Additionally, the FR-OIF scheme is also able to address accurate localization in the presence of sensor failures or erroneous measurements. To demonstrate the effectiveness of the proposed fusion architecture, extensive experimental studies have been conducted with a micro aerial vehicle, equipped with various onboard pose sensors, such as a 3D lidar, a real-sense camera, an ultra wide band node, and an IMU. The efficiency of the proposed novel framework is extensively evaluated through multiple experimental results, while its superiority is also demonstrated through a comparison with the classical multi-sensorial centralized fusion approach.

## 1. Introduction

State estimation is a challenging problem in the field of robotics that has been significantly explored in the recent years and in different scientific and technological oriented communities, such as: robotics [1], aerospace [2], automatic control [3], artificial intelligence [4], and computer vision [5]. In this framework, one of the most interesting problems is the one that is related to the estimation of the pose of a robot, especially for the case that multi-sensors are utilized for the pose determination problem, with related sensor fusion schemes, in order to increase the overall accuracy of the estimation but also at the same time introduce the proper resiliency.

Towards this direction, lately the research on sensor fusion has been evolving in a rapid manner, since multi-sensor fusion has the ability to integrate or to combine data streams from different sources and simultaneously to increase the quality of the measurements, while decreasing the corrupting noise from the measurements. Thus, in the multi-sensorial fusion architectures, such for example the case of the pose estimation, it is very common to have multiple sensors that are providing, e.g., full pose state estimations or partial pose estimations (translation or orientation) and to have an overall sensor fusion scheme that is commonly realized in a centralized [6] or distributed approaches [6,7]. In centralized fusion architectures, a central node or a single node is utilized, where direct measurement data or raw data from multiple sensors are used to fuse using several type of Kalman filters, depending upon the system whether it is linear or nonlinear. In this context, the Extended Kalman Filter (EKF) has taken considerable attention in numerous research works involving the case of multi-sensor fusion, while utilizing real data from sensors for localization of ground robots and Micro Aerial Vehicle (MAV) [8,9,10,11]. However, the EKF based fusion algorithms involve local linearization of the system. In order to explicitly account for the nonlinearities, involved in the dynamical model, few progressive approaches consider the Unscented Kalman Filter (UKF) [12,13] and the Particle Filter (PF) [14] based multi-sensor fusion for robotic localization. Even though the UKF and PF are superior for addressing nonlinearities, the advantage comes with additional complexity in the computational burden. Comparison studies reported in [15,16] demonstrated that for robot localization, the performance of EKF is comparable in all practical purposes. Apart from the conventional Kalman based approaches, various innovative numerical optimization based multi-sensor fusion algorithms, such as moving horizon estimation [17,18], set membership function [19], graph optimization [20], while MAV localization in GPS denied environment have recently been investigated in [19,21]. The numerical optimization based estimation framework has the capability to incorporate the non-linearity of the dynamical model, as well as various physical constraints. However, it imposes additional computation complexity and limitations due to the theoretical guarantee on convergence properties.

In general, a centralized structure with multi inputs and outputs works very well for data fusion. Though it is not sufficient for all the cases, sometimes one of the sensor can fail suddenly for a certain period of time in total operating duration, in such a situation, the centralized approach follows the faulty sensor’s data, while having the disadvantage that it can not detect or eliminate the fault occurred by the sensors. Although it is possible to attain an almost optimal solution in the centralized fusion framework, in real and field utilizations, processing all the sensors at a node or a point is most likely ineffective and has the potential to lead into direct failures in case of a sensor defects or temporary performance degradation. On the other hand, the distributed fusion [22] typically consists of at least two layers, where in the first layer, the raw data are collected from different sensor measurement units to create the local estimates and in the sequel are being forwarded to the second layer for further fusion from the corresponding nodes. Typically, in the first layer a Kalman filter is utilized to provide the local estimates from the sensors [23]. However, depending on the data assimilation procedures, in the second layer various decentralized fusion methods have been investigated [24]. In this direction, an information filter based decentralized fusion [22] is considered for the localization of mobile robots and MAVs in [24,25]. However, these formulations do not explicitly incorporates the correlation between local estimates. Contemplating the dependency of the local nodes, various covariance intersection based decentralize fusion algorithms for collaborative localization have been investigated in [26,27,28,29,30]. In this context, an innovative maximum likelihood criterion for performing the decentralized fusion was presented in [31], where the information from the local nodes were integrated as a weighted combination to provide the fused states. The weighting matrices were judiciously determined based on the cross covariance of the local estimates. However, these decentralized fusion approaches, have been implemented based on the assumption that the measurements from sensors are only influenced by an inherent unbiased Gaussian noise, while in reality, and for field robotic applications, the related measurements are spurious [30] due to unexpected uncertainties, such as a temporal inoperative surrounding (such as low illumination condition, presence of smoke/dust etc.) fault, spike and sensor glitches. In such situations, the magnitude of inaccuracy in the measurements is much larger when compared to the normal noise [32]. In order to address this issue, various fault detection and isolation methods, in combination with the decentralized fusion, are investigated in the related state of the art literature [28,33,34].

In summary, distributed fusion is a robust framework to failures and can indicate the proper resiliency for critical applications, while eliminating the risk of single failures. Specifically in robotic applications, the approach of perception, in the direction of decentralized fusion, can handle the fundamental problem addressed in numerous implementations like multi-robot tracking, cooperative localization [35] and navigation [35], multi-robot Simultaneous Localization and Mapping (SLAM) [36], distributed multi-view 3D reconstruction and mapping, and multi-robot monitoring [37]. The map merging, data association, and robot localization can potentially be efficient only if the robots are enough capable to perceive autonomously the world. Therefore, centralized and decentralized fusion or in other words fusion in general, plays an important role in all robotic applications.

In this article, a unique decentralized multi-sensor fusion approach is proposed that introduces a novel flexible fault resilient structure, by isolating the information from the faulty sensor, while enabling the information provided by the other sensor measurements in an optimal information fusion framework. Furthermore, the classical optimal information fusion (OIF) presented in [31] has a provision to incorporate a sensor level fault isolation, which operates as a separate unit. However, an increasing number of sensors/local nodes, along with the presence of temporal sporadic (spikes, temporary failure) measurements, demands a more flexible fusion architecture to account for fault resiliency in real time. In the present work, an innovative fault isolation mechanism is embedded with the OIF architecture by representing the isolation operation, as a constraint optimization problem. Thus, the novelty of this article stems from the novel application of an optimal information two-layered sensor fusion method, which has been modified ideally for handling sensor breakdown during operation, as it will be described in the sequel.

The main focus of this article is to develop a two-layered, multi-sensor fusion with self resiliency with the assistance of a unique optimal isolation algorithm. The technique works in several stages. In the first step, information from multiple asynchronous sensors is exhaustively exploited in an orderly sequence by introducing the concept of nodes. Each node individually fuses position and orientation information from independent sensors separately. In a way, the nodal architecture introduces the feasibility of distinguishing partially defective measurements and thereby broaden out the possibility of fusing all the acceptable information. For instance, if a sensor provides accurate position and defective orientation measurement for a short duration, utilization of the position measurement would be beneficial in lieu of discarding the entire sensor information. Next, in the second layer, the information from each node is blended in a weighted combination by employing an maximum likelihood estimator to obtain the most accurate pose collectively. Moreover, the proposed fault resilient optimal information fusion architecture incorporates an inbuilt fault isolation mechanism to discard disputed outcome from sensors. Once the disputed outcomes are observed, the weighing parameters of the optimal information filter are adjusted to accommodate the fault isolation. The second contribution stems from the effectiveness evaluation of the second layer fusion architecture when a sensor is not working correctly for a certain period or when the system receives inaccurate measurements from the sensors. In this case, an optimal information filter with fault handling capability is proposed and incorporated to get more robust and accurate responses from the corrupted outcomes. In this case, a modified co-variance weight scheme is introduced for combining all possible nodes in the second layer that is utilized to resolve the effect of erroneous measurements. The final contribution of the article is towards the performance comparison of the existing centralized and decentralized fusion techniques with the proposed novel decentralized fusion approach. Both existing methods methods perform well when measured sensor data are faultless, otherwise, the proposed fault resilient decentralized fusion scheme works far better than the existing centralized and decentralized filter estimation. The comparison of these different fusion architectures ha been carried out by using experimentally collected data sets.

## 2. Problem Formulation

**Figure 1:**
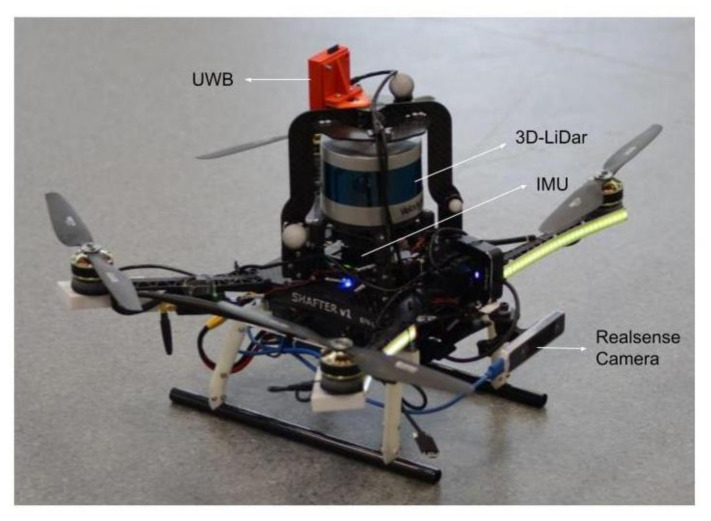
The aerial robot considered with the heterogeneous and asynchronous sensors for establishing the multi-layer sensor fusion architecture.

**Figure 2:**
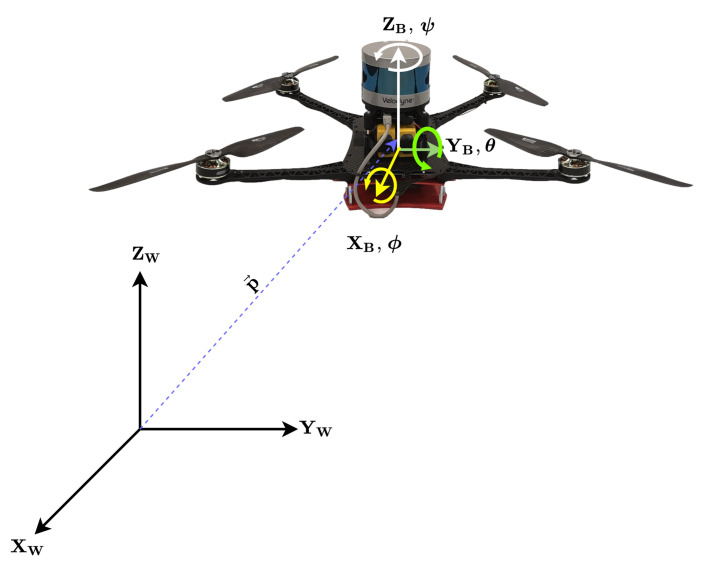
Co-ordinate frames: subscript *W* denotes the global frame and subscript *B* denotes the body frame.

Aiming towards a real-time, resilient and accurate navigation for autonomous exploration, through an unknown environment, aerial robotic vehicles equipped with multiple asynchronous real senors, as the one depicted in Figure 1 will be considered as the base line of this novel work, without a loss of generality, since the overall framework has the merit to be platform agnostic. In this case, the considered sensor suite of the MAV contains a Velodyne Puck LITE, the Intel realsense camera T265, the IMU of a Pixhawk 4 flight controller, and a single UWB node. The collected point-cloud from the 3D-lidar is processed online to provide odometry based on [38]. Essentially, the 3D-lidar odometry and the real sense camera T265 are capable of providing the real time 3D pose of the MAV independently, whereas, the IMU provides the measurements of angular velocity, as well as the acceleration of the MAV. In addition, a network of 5 UWB nodes is set strategically around the utilized flying arena, for estimating the position of the MAV based on the mounted UWB node. The overall objective is to provide a novel decentralized multi-sensor fusion architecture that can blend the information from various real sensor measurements to obtain the most accurate pose of the MAV in real-time. A schematic representation of the MAV pose is depicted in Figure 2.

The pose is described using two reference frames, namely the world frame $\left(W=\left\{X_{W},Y_{W},Z_{W}\right\}\right)$ and the body-fixed frame $\left(B=\left\{X_{B},Y_{B},Z_{B}\right\}\right)$. The body-fixed frame is attached to the MAV’s centre of mass, while the inertial frame is assumed to be attached at a point on the ground with its X,Y and *Z* axis directed along the East, North and Up (ENU) directions, respectively. The sensors are mounted in the body fixed frame B of the MAV, and provide the information regarding its pose. The position of the MAV (essentially the origin of the body-fixed frame) is defined as $\vec{p}$, which is described with respect to the world frame W as shown in Figure 2. The orientation of MAV with respect to the world frame can be visualized using the Euler angle representation {ϕ,θ,ψ}, denoting the roll, pitch and yaw rotations, respectively. In order to avoid the singularity associated with Euler angle, the orientation of the MAV is considered to be represented by quaternions [39] denoted with *q*. The position of the MAV, *p* and its orientation, *q* together designate the pose of the vehicle.

### 2.1. Asynchronous Sensors Associated with the Present Study

The under consideration MAV, equipped with various sensors, is presented in Figure 1. The sensors that are considered in the present context are: (a) the IMU of a Pixhawk 4 flight controller that provides the acceleration $a_{m}$, the angular velocity $\omega_{m}$ and the orientation $q_{IMU}$ of the MAV, (b) the Velodyne Puck LITE radar assisted with Lidar Odometry (LIO) [38] to provide a 3D pose of the MAV denoted by $p_{LIO},q_{LIO}$, (c) the Intel Real-sense T265 visual sensor integrated with visual odometry (VIO) that provides a position and orientation denoted with $p_{VIO},q_{VIO}$, and (d) the Ultra-Wideband (UWB) transceivers that provide the position of the MAV, denoted with $p_{UWB}$.

### 2.2. The MAV’s Utilized Kinematic Model

The decentralized sensor fusion architecture at its core utilizes the model based estimation framework, where the nonlinear kinematic model of the MAV is considered as [40]:

(1a) $$\begin{array}{cc} \; & \dot{p}=v_{t} \end{array}$$

(1b) $$\begin{array}{cc} \; & {\dot{v}}_{t}=a_{t} \end{array}$$

(1c) $$\begin{array}{cc} \; & \dot{q}=\frac{1}{2}q⊗\omega_{t} \end{array}$$

where, $p\in R^{3\times 1}$ denotes the position, $v_{t}\in R^{3\times 1}$ represents the velocity, $q\in R^{4\times 1}$ stands for the orientation of the MAV in the form of quaternions representation. Here, $a_{t}\in R^{3\times 1}$ represents the total acceleration of the vehicle expressed in the inertial frame of references, where as $\omega_{t}\in R^{3\times 1}$ denotes the body rate experienced by the vehicle. Physically, these parameters (acceleration and body rates) are characterized as an input to the system, which are typically measured using an IMU denoted as $a_{m}$ and $\omega_{m}$. In general, the measurements from sensors are noisy, which includes sensor bias as well. In order to establish this fact, the measurement signals are represented as:

(2a) $$\begin{array}{cc} \; & a_{m}=R_{t}^{T}\left(a_{t}-g_{t}\right)+a_{b_{t}}+a_{n} \end{array}$$

(2b) $$\begin{array}{cc} \; & \omega_{m}=\omega_{t}+\omega_{b_{t}}+\omega_{n} \end{array}$$

where, $a_{b_{t}}\in R^{3\times 1}$ denotes the accelerometer bias and $\omega_{b_{t}}\in R^{3\times 1}$ represents the gyroscopic bias terms, $a_{n}$ and $\omega_{n}$ signify the additive noise for acceleration and angular rate, respectively, $R_{t}≜R\left(q\right)\in SO\left(3\right)$ denotes the transformation matrix, from the body to the world frame, and $g_{t}\in R^{3\times 1}$ denotes the gravity bias. Moreover, it has also been accounted that the bias factors $a_{b_{t}},\;\omega_{b_{t}}$ are driven by a process noise dynamically represented as:

(3a) $$\begin{array}{cc} {\dot{a}}_{b_{t}} & =a_{\omega } \end{array}$$

(3b) $$\begin{array}{cc} {\dot{\omega }}_{b_{t}} & =\omega_{\omega } \end{array}$$

(3c) $$\begin{array}{cc} {\dot{g}}_{t} & =0_{3\times 1} \end{array}$$

where, $a_{\omega }\in R^{3\times 1}$ and $\omega_{\omega }\in R^{3\times 1}$ are the accelerometer and gyroscopic process noise, respectively. Rearranging Equation (4a,b), the total acceleration can be expressed as:

(4a) $$\begin{array}{cc} a_{t} & =R_{t}\left(a_{m}-a_{b_{t}}-a_{n}\right)+g_{t} \end{array}$$

(4b) $$\begin{array}{cc} \omega_{t} & =\omega_{m}-\omega_{b_{t}}-\omega_{n} \end{array}$$

Substituting, Equations (4a,b) and (1b,c), respectively, yields:

(5a) $$\begin{array}{cc} {\dot{v}}_{t} & =R_{t}\left(a_{m}-a_{b_{t}}-a_{n}\right)+g_{t} \end{array}$$

(5b) $$\begin{array}{cc} \dot{q} & =\frac{1}{2}q⊗\left(\omega_{m}-\omega_{b_{t}}-\omega_{n}\right) \end{array}$$

The above equation of motion of MAV is expressed in a compact mathematical notation given as:

(6a) $$\begin{array}{cc} {\dot{X}}_{t} & =f_{t}\left(X_{t},u_{m},w\right) \end{array}$$

(6b) $$\begin{array}{cc} y_{t} & =\left[I_{7\times 7}|0_{7\times 12}\right]X_{t} \end{array}$$

where, the state vector is denoted as $X_{t}={\left[p_{t},v_{t},q,a_{bt},\omega_{bt},g_{t}\right]}^{T}\in R^{19\times 1}$, the noisy measured input based on IMU reading is denoted as $u_{m}\in R^{6\times 1}$ and the random process noise $w\in R^{6\times 1}$ is defined as:

$$u_{m}=\left[\begin{array}{c} a_{m}-a_{n}\\ \omega_{m}-\omega_{n} \end{array}\right]\;,\;w=\left[\begin{array}{c} a_{w}\\ \omega_{w} \end{array}\right]$$

## 3. Decentralized Sensor Fusion Architecture

**Figure 3:**
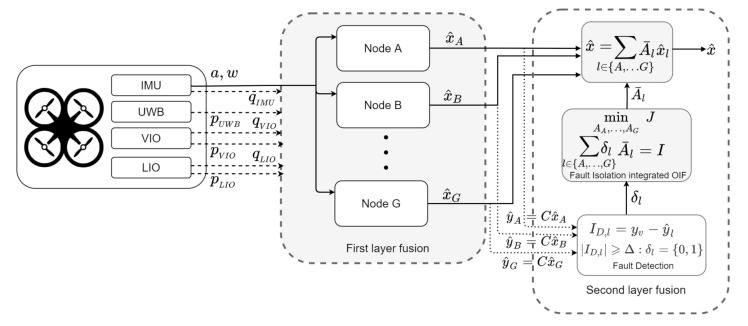
The Fault resilient optimal information filter with a two-layered fusion arrangement. An emphasized description of the first layer fusion is given in Figure 4.

In order to make use of all the available information from the sensor measurements in the best possible way, a two-layered fusion architecture is considered here. A schematic overview of the proposed fusion architecture is presented in Figure 3. Since the primary focus here is to determine the MAV pose, the sensors involved in this process are capable of measuring either the position and/or orientation, while the IMU is the only exception, which provides the acceleration and body rates of the vehicle. In the First layer, information from multiple asynchronous sensors is exhaustively exploited in an orderly sequence by introducing the concept of nodes. Potentially, each node provides the pose of the MAV, which is obtained by involving its position from one sensor and orientation from another. In the second layer, the information from each node is used in a weighted combination to collectively obtain the most accurate pose of the MAV in a maximum likelihood hood manner. A complete overview of the fusion architecture in detail is presented in the sequel.

### 3.1. First Layered Decentralized Fusion Architecture

**Figure 4:**
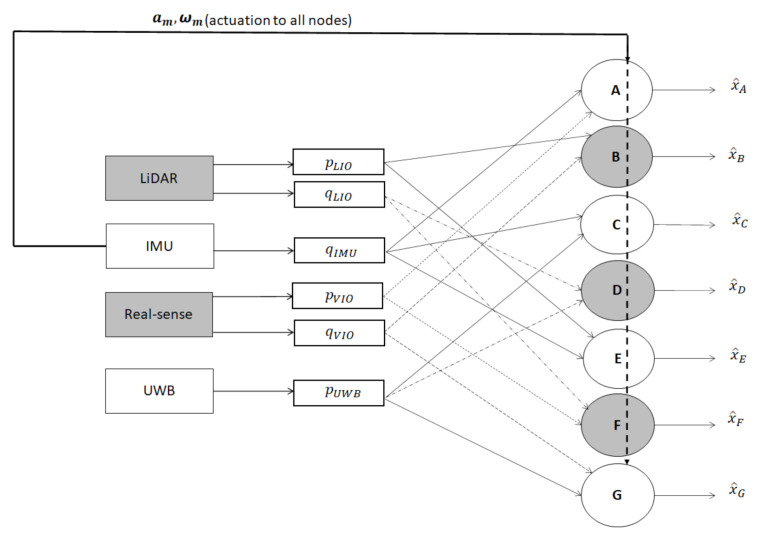
Combination of position and orientation information flow from various sensors in constituting different nodes of the first layer.

In the first layer, we have introduced the concept of the decentralized nodes. In the context of the multi-sensor framework, the position and orientation of the MAV is obtained by two distinct sensors that are arbitrarily selected to construct a node. Incorporating all of such possible combinations of sensor measurements collectively, a total number of seven nodes are constructed in the present setup and alphabetically denoted as node-*l*, where l∈{A,B,⋯,G}. The position and orientation information accounted for the individual nodes are described in Table 1. In the process of constructing the node, each decentralized node is associated with an Extended Kalman Filters (EKF) [41], which blends the measurements from various sensors in all possible combinations. However, each node receives the IMU information, as measured actuation/control input to the kinematic model associated with the Kalman filter. Moreover, position and orientation from two distinct sensors are utilized as the measurement information. In the context of the present setup under consideration, Figure 4 describes all the possible decentralized nodes and the associated measurements [42].

Previously, in the Section 2.2, the kinematic model of a MAV in continuous time form is presented in Equation (6a). In order to describe the decentralized nodes, associated with the first layer, in a compact mathematical form (in correlations with EKF), an equivalent of Equation (6b) in discrete time representation (using Euler [43] discretization) is provided by:

(7a) $$\begin{array}{cc} x_{k} & =f_{k-1}\left(x_{k-1},u_{k-1},\omega_{k-1}\right) \end{array}$$

(7b) $$\begin{array}{cc} \;y_{k} & =h_{k}\left(x_{k},v_{k}\right)=\left[I_{7\times 7}|0_{7\times 12}\right]x_{k}+v_{k} \end{array}$$

where, *k* denotes the discrete time instants. It should be noted that in order to account for model inaccuracy, we have considered a process noise $\omega_{k}\in R^{19}$. Moreover, an additional measurement noise vector $v_{k}\in R^{7}$ is introduced in Equation (7b) to encapsulate a realistic output model, under the influence of noisy measurement appearing from real sensors. The process and measurement noise are assumed to follow the Gaussian distribution as:

(8) $$\begin{array}{c} \omega_{k}∼N\left(0,\;Q_{k}\right),v_{k}∼N\left(0,\;R_{k}\right)\; \end{array}$$

where $Q_{k}$ and $R_{k}$ represents the process noise co-variance matrix and the measurement noise co-variance matrix, respectively. The mathematical operator *E* denotes the expectation and the superscript *T* indicates the transpose. Starting with an initial guess of a posteriori estimate ${\hat{x}}_{l_{0}}^{+}=E\left(x_{l_{0}}\right)$ and $P_{l_{0}}^{+}=E\left[\left(x_{l}-{\hat{x}}_{l_{0}}^{+}\right){\left(x_{l}-{\hat{x}}_{l_{0}}^{+}\right)}^{T}\right]$, along with the assumption in Equation (8), the $l^{\mathrm{th}}$ node is described as a local EKF with the following prediction-correction formalism: 

*Prediction Steps:*

(9a) $$\begin{array}{cc} {\hat{x}}_{l_{0}}^{+} & =E\left(x_{l_{0}}\right),P_{l_{0}}^{+}=E\left[\left(x_{l}-{\hat{x}}_{l_{0}}^{+}\right){\left(x_{l}-{\hat{x}}_{l_{0}}^{+}\right)}^{T}\right] \end{array}$$

(9b) $$\begin{array}{cc} {\hat{x}}_{l_{k}}^{-} & =f_{l_{k-1}}\left({\hat{x}}_{l_{k-1}}^{+},u_{k-1},0\right) \end{array}$$

(9c) $$\begin{array}{cc} K_{l_{k}} & =P_{l_{k}}^{-}H_{l_{k}}^{T}{\left(H_{l_{k}}P_{l_{k}}^{-}H_{l_{k}}^{T}+R_{k_{l}}^{T}\right)}^{-1} \end{array}$$

(9d) $$\begin{array}{cc} P_{l_{k}}^{-} & =F_{l_{k}}P_{l_{k}}^{+}F_{l_{k}}^{T}+L_{l_{k}}Q_{l_{k}}L_{l_{k}} \end{array}$$

where the ‘+’ symbol is used to denote an a priori estimate, the ‘−’ symbol is designated to a posteriori estimate, the subscript *l* indicates the corresponding variable of the *l*th node, where l∈{A,B,⋯,G}. The Jacobian matrices are defined as:

(10) $$\begin{array}{c} F_{l_{k}}=\frac{\partial f_{l_{k-1}}}{\partial x_{l_{k}}},\;L_{l_{k}}=\frac{\partial f_{l_{k}}}{\partial u_{k}},\;H_{l_{k}}=\frac{\partial h_{l_{k}}}{\partial xl_{k}} \end{array}$$

while the inputs excitation $u_{k}$ (linear acceleration and angular velocity), used in the prediction process, are essentially obtained from the IMU measurements. 

*Correction Steps:*

(11a) $$\begin{array}{cc} {\hat{x}}_{l_{k}}^{+} & ={\hat{x}}_{l_{k}}^{-}+K_{l_{k}}\left[y_{l_{k}}-h_{l_{k}}\left(x_{l_{k}}^{-},0\right)\right] \end{array}$$

(11b) $$\begin{array}{cc} \;P_{l_{k}}^{+} & =\left(I-K_{l_{k}}H_{l_{k}}\right)P_{l_{k}}^{-} \end{array}$$

Note that, in the process of constructing the nodes, the associated EKF cleans out the noisy measurements appearing from the actual sensor unit. It is apparent that, individually each node is potentially capable of proving the information regarding the pose of the MAV. However, the accuracy of pose information, obtained from each decentralized node, varies depending on the accuracy of the sensors that are involved to constitute the node. Hence, to obtain a best possible pose estimate, a second layer architecture is presented in the sequel.

### 3.2. Second Layer Decentralized Fusion Architecture

The estimated states ${\hat{x}}_{l}$, l∈{A,B,⋯,G} from each node are placed together in a weighted combination to jointly obtain an accurate estimate of the MAV pose, while using an Optimal Information Filter (OIF) by maximum likelihood estimation. Thus, the FR-OIF is used in the second layer, which incorporates the capability of choosing optimal weights based on the co-variances that is obtained from the first layer fusion. Moreover, the proposed formulation embeds a fault isolation mechanism with the OIF architecture. The collective estimate of the fused state vector, as the output of the second layer, is expressed as:

(12) $${\hat{x}}_{k}=\sum_{l\in \{A,\cdots ,G\}}{\bar{A}}_{l_{k}}\hat{x_{l_{k}}}$$

where, ${\bar{A}}_{l_{k}},l\in \left\{A,\;B,\cdots ,G\right\}$ represents the arbitrary weight associated with the corresponding node. Here ${\hat{x}}_{k}$ and ${\hat{x}}_{l_{k}}$ denote the outcome of the second layered fusion and estimated states of the $l^{\mathrm{th}}$ node from the first layer, respectively. These weigh parameters are optimally determined based on a minimum variance (maximum likelihood) criterion.

Assuming that both the FR-OIF, as well as the EKF act as an unbiased estimator, i.e., $E\left({\hat{x}}_{k}\right)=E\left(x_{k}\right)$, $E\left({\hat{x}}_{l_{k}}\right)=E\left(x_{k}\right)$, and taking the expectation of both sides of Equation (12) it yields:

(13) $${\bar{A}}_{A_{k}}+{\bar{A}}_{B_{k}}+......+{\bar{A}}_{G_{k}}=I$$

where $x_{k}$ represents the actual state of the MAV. The estimation error for FR-OIF, i.e., $x_{k}-{\hat{x}}_{k}$ is expressed as:

(14) $${\tilde{x}}_{k}=x_{k}-\sum_{l\in \{A_{k},\cdots ,G\}}{\bar{A}}_{l_{k}}{\hat{x}}_{l_{k}}$$

Using the constraint relation from Equation (13), the estimation error in Equation (14) is rewritten as:

(15) $$\tilde{x_{k}}=\sum_{l\in \{A,\cdots ,G\}}\bar{A_{l_{k}}}\left(x_{k}-{\hat{x}}_{l_{k}}\right)=\sum_{l\in \{A,\cdots ,G\}}{\bar{A}}_{l}{\tilde{x}}_{l_{k}}=W_{k}{\tilde{x}}_{L}$$

where $W_{k}={\left[{\bar{A}}_{A_{k}},{\bar{A}}_{B_{k}},\cdots {\bar{A}}_{G_{k}}\right]}^{T}$ and ${\tilde{x}}_{L}=\left[{\tilde{x}}_{A_{k}},{\tilde{x}}_{B_{k}},\cdots ,{\tilde{x}}_{G_{k}}\right]$. Hence, the error co-variance matrix of the second layer of the FR-OIF is expressed as:

(16) $$P_{k}=E\left({\tilde{x}}_{k}{\tilde{x}}_{k}^{T}\right)=W_{k}^{T}\Sigma_{k}W_{k}$$

where $\Sigma_{k}=E\left({\tilde{x}}_{L}{\tilde{x}}_{L}^{T}\right)=P_{{\left(l,m\right)}_{k}},\forall l=m=\left\{A,B,\cdots ,G\right\}$ represents the cross co-variance matrix [31] between *l*th and *m*th node, expressed as:

(17) $$P_{{\left(l,m\right)}_{k}}^{-}=\left[I-K_{m_{k}}H_{m_{k}}\right]\times \left[F_{l_{k}}P_{{\left(l,m\right)}_{k}}^{+}F_{l_{k}}^{T}+Q_{k}\right]\times {\left[I-K_{m_{k}}H_{m_{k}}\right]}^{T}$$

At this point, it is possible to obtain the weight parameter matrix *W* by solving the following static optimization, as described in [31]:

(18) $$\begin{array}{cc} \;\; & \min_{A_{A_{k}},\cdots ,A_{G_{k}}}\;J_{k}=\frac{1}{2}tr\left(P_{k}\right)=\frac{1}{2}tr\left(W_{k}^{T}\Sigma_{k}W_{k}^{T}\right)\\ \; & \mathrm{subjected}\;\mathrm{to}\;\left({\bar{A}}_{A_{k}}+{\bar{A}}_{B_{k}}+.......+{\bar{A}}_{G_{k}}\right)=I \end{array}$$

However, the solution of Equation (18), obtained from the classical OIF in [31], is unable to provide a sufficient resiliency in the presence of inaccurate measurements obtained from one or a group of sensors. However, such problems of corrupted sensor measurements for a short duration of the operation period are often encountered in reality. For example, in the absence of sufficient visual features for some part of the surrounding environment, the real-sense camera fails to determine the MAV’s position, or in the presence of a bright moving object in the lidar’s field of view, it fails to provide an accurate pose.

In order to overcome such shortcomings, a separate fault isolation technique is proposed as an enhancement to the proposed decentralized estimation scheme, where the classical OIF formulation is modified to incorporate an inbuilt fault isolation mechanism with the OIF structure. For a time interval, if a group of sensors is identified to be corrupted, based on a fault detection method, the corresponding nodes associated with the faulty sensor need to be eliminated from the second layer architecture during the defective period of operation. In this article and without a loss of generality, the mechanisms for identifying the fault occurrence will not be considered and it will be assumed that the time of the fault and the faulty node can be identified. However, a straightforward nullifying of the weight matrices linked with the corrupted nodes, without altering other weights, leads to violation of the constraint in Equation (13). Hence, it is required to reformulate the optimization problem to bring in the flexibility of enabling/disabling a group of nodes online, while the MAV is operating in real-life applications. Let us consider the *i*th node, where $i\in A_{k},B_{k},\cdots ,G_{k}$, is found to be corrupted for a short time interval. This brings an additional constraint collectively presented as:

(19) $$\sum_{l\in \{A,\cdots ,G\}}{\bar{A}}_{l_{k}}=I,\;\;\;\;\;\;\sum_{i\in \{A,\cdots ,G\}}{\bar{A}}_{i_{k}}=0,\;\;\;\;\;\;i\notin l$$

The above constraints are combined and with a compact mathematical notation and can be represented as:

(20) $$\sum_{l\in \{A,\cdots ,G\}}\delta_{l_{k}}{\bar{A}}_{l_{k}}=I$$

where, $\delta_{l_{k}}\in \left\{0,1\right\}$ is a scalar multiplying factor. We will impose that $\delta_{l_{k}}=0$, if the *l*th node is found to be corrupted, otherwise $\delta_{l_{k}}=1$. The modified optimization problem is presented as:

(21) $$\begin{array}{cc} \; & \min_{A_{A},\cdots ,A_{G}}\;J_{k}=\frac{1}{2}tr\left(P_{k}\right)=\frac{1}{2}tr\left(W_{k}^{T}\Sigma_{k}W_{k}^{T}\right)\\ \; & \mathrm{subjected}\;\mathrm{to}\;\left(W_{k}^{T}e_{\delta_{k}}-I\right)=0 \end{array}$$

where, $e_{\delta_{k}}={\left[\delta_{A_{k}}I,\;\delta_{B_{k}}I,\;\cdots ,\;\delta_{G_{k}}I\right]}^{T}$. Following the solution approach of the optimization problem with an equality constraint using the Lagrange multiplayer method [44], the augmented cost function is presented as:

(22) $${\bar{J}}_{k}=\frac{1}{2}tr\left(W_{k}^{T}\Sigma_{k}W_{k}\right)+tr\left[\Lambda_{k}\left(W_{k}^{T}e_{\delta_{k}}-I\right)\right]$$

where, $\Lambda_{k}\in R^{19\times 19}$ represents the Lagrange multiplayer. Evaluating the necessary conditions of optimality, i.e., $\frac{\partial {\bar{J}}_{k}}{\partial W_{k}}=0$ and $\frac{\partial {\bar{J}}_{k}}{\partial \Lambda_{k}}=0$, yields:

(23) $$\left[\begin{array}{cc} \Sigma_{k} & e_{\delta_{k}}\\ {e_{\delta_{k}}}^{T} & 0 \end{array}\right]\left[\begin{array}{c} W_{k}\\ \Lambda_{k} \end{array}\right]\;=\;\left[\begin{array}{c} 0\\ I \end{array}\right]$$

From the solution of Equation (23), the optimal weight matrix $W_{k}$ is obtained as:

(24) $$W_{k}=\Sigma_{k}^{-1}e_{\delta_{k}}{\left({e_{\delta_{k}}}^{T}\Sigma_{k}^{-1}e_{\delta_{k}}\right)}^{-1}$$

Using the optimal weight matrix $W_{k}$ into Equation (12), one can obtain the estimated state from the second layer fusion architecture. The revised $W_{k}$, as a function of $e_{\delta_{k}}$, enables the modified OIF to be resilient in presence of a faulty measurement from a group of sensors.

#### Fault Detection

In the presented methodology, the corruption or the fault of a sensor is diagnosed by the first layer fusion, while the Kalman filter innovation from Equation (11a) is utilized to detect the fault or locate the sensor failure at the corresponding time frame. The innovation can be written as $I_{D,l}=\left[y_{v_{k}}-h_{k}\left({\hat{x}}_{k},0\right)\right]$. In this way, the innovation vector $I_{D,l}$ depends on the estimated poses from the individual nodes with the pose obtained from a Vicon motion capturing system. In this case the sub-scripted letter *D* denotes the detected fault, while the sub-scripted letter *v* indicates the pose obtained from a Vicon. Furthermore, Vicon provides the most accurate pose, which can be considered as a ground truth. Once the innovation for all the nodes is computed, it is compared with a threshold value. The logical rule is given as:

(25) $$\left\{\begin{array}{c} \left|I_{D,l}\right|⩾\Delta :\;\;\delta_{l}=0,lth\mathrm{node}\mathrm{is}\mathrm{faulty}\\ 0\leq \left|I_{D,l}\right|<\Delta :\delta_{l}=1,lth\mathrm{node}\mathrm{is}\mathrm{legitimate} \end{array}\right.$$

The fault $f_{l}$ occurs. $\left|.\right|$ is denoted for absolute value operator and Δ is the threshold that can be chosen arbitrary.

## 4. Experimental Framework Evaluation

For the experimental evaluation of the proposed scheme, collected data from a MAV under a manual flight is utilized. The platform and its components have been depicted in Figure 1. In this case, the sensor suite of the MAV consists of the Velodyne Puck LITE based Lidar odometry, the intel real-sense camera T265 for visual odometry, the IMU of a Pixhawk 4 flight controller, and a single UWB node. The detail about the sensor suit is described in Section 2. The MAV is manually flown in an approximate rectangular trajectory. During the experiment, information from the multiple sensors are recorded, which is used to evaluate the efficacy of the proposed FR-OIF framework. Apart form the on-board sensor suit, a Vicon motion capture system is used to provide the most accurate pose of MAV, which is considered as the ground truth in the present context.

The fusion method works in three different stages when sensor outcomes are faulty. In the first step, various nodes generate their equivalent estimated states and associated innovation (as presented in Equation (11a)). The actuation input (Angular velocity $\omega_{m}$ and linear acceleration $a_{m}$) for all the nodes are obtained from IMU, which is shared among all the nodes. In the second step, innovation terms for various nodes are compared with a constant threshold. The process essentially identifies the defective node. Eventually, in the last effort, the resilient fault isolation mechanism of FR-OIF architecture eliminates the faulty measurements. The corresponding numerical values for the various systems and design parameters that are considered in the present article are: initial guess for error co-variance $P_{l_{0}}=I_{19\times 19}$, initial state $X_{l_{0}}={\left[0_{1\times 6},1,0_{1\times 10},9.81\right]}^{T}\forall l\in \left(A,\cdots ,G\right)$, process noise co-variance $Q_{l_{k}}=1000\times I_{19\times 19}$, measurement noise co-variance $R_{l_{k}}=10\times I_{18\times 18}$, threshold tolerance for innovation Δ=0.4. The experimental results, along with a comparison study based on the centralized EKF approach will be presented in the sequel.

**Figure 5:**
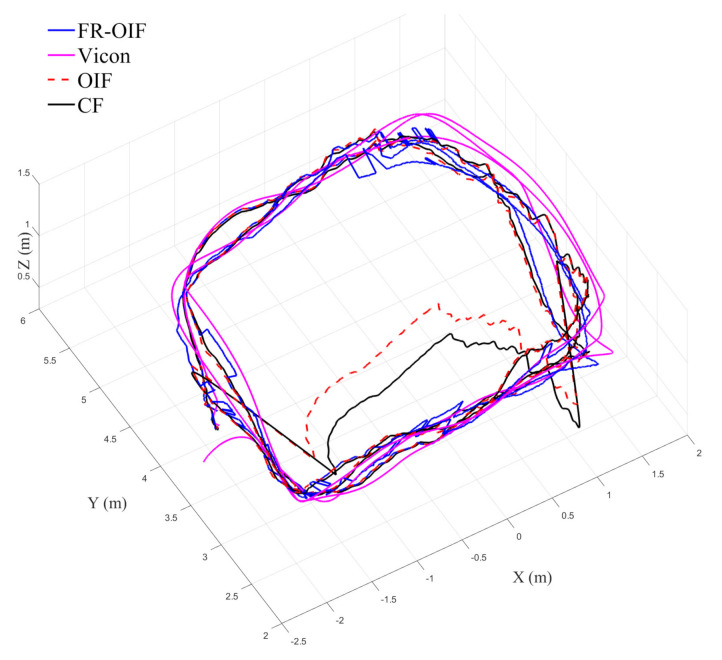
Variation of estimated MAV trajectory obtained from Centralized Fusion (CF), decentralized Optimal Information Fusion (OIF), Fault Resilient (FR)-OIF, and ground truth (position from vicon camera). FR-OIF provides the estimated position of the MAV approximately close with the ground truth obtained from the Vicon motion capture system.

**Figure 6:**
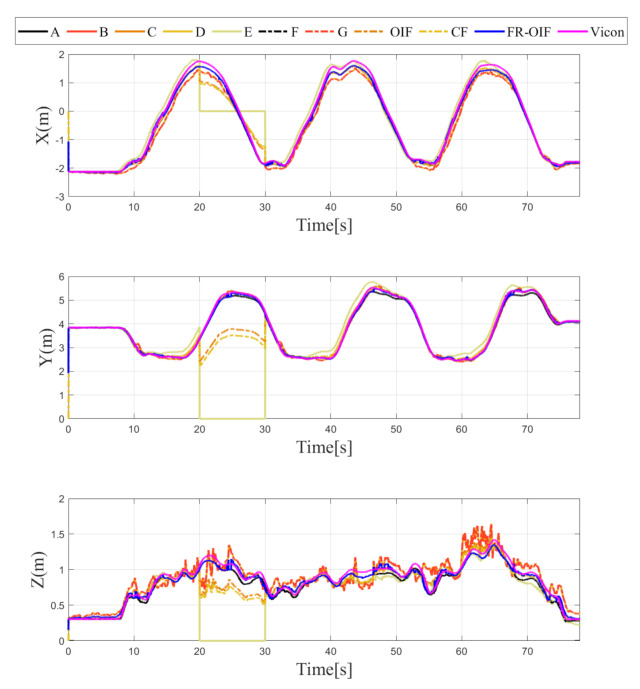
Estimated positions along ‘X-Y-Z’ components obtained from intermediate nodes (A,⋯,G) and FR-OIF, compared with CF, OIF and the ground truth. The evaluation is carried out in presence of temporal fault appearing from LIO (Case-1). Except the fused position obtained from FR-OIF all the estimated positions deviated during 20–30 s.

**Figure 7:**
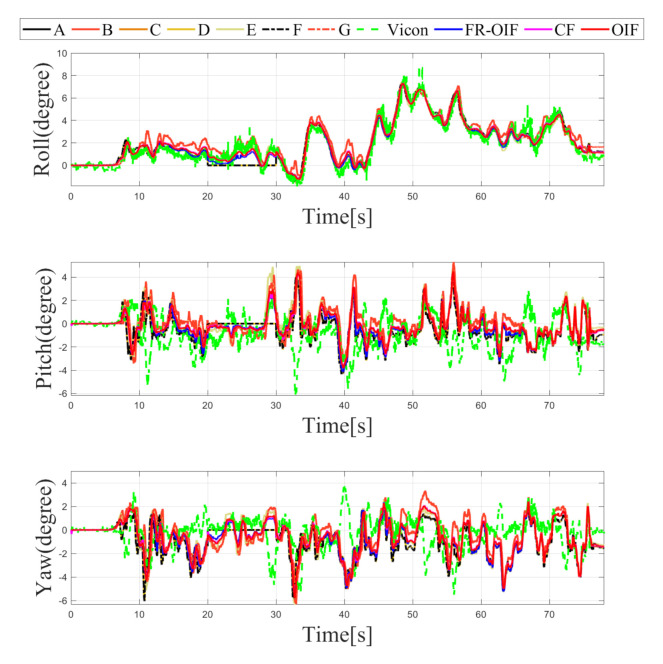
Variation of orientation represented using Euler angles obtained from intermediate nodes (A,⋯,G), CF, OIF, FR-OIF and the ground truth. During the experiment, the transnational motion is dominant over the rotational motion. As a result, the significant impact of FR-OIF is difficult to be visualized.

The estimated trajectory of the MAV is presented in Figure 5. From the obtained results, it is evident that the FR-OIF provides a pose estimate that is approximately close to the ground truth trajectory obtained from the Vicon system. The variation of the estimated MAV position along X,Y,Z are presented in Figure 6. It can be observed in Figure 5 that, in the absence of faulty measurement, the estimated trajectory obtained from the Centralized fusion (CF) as well as the decentralized Optimal information fusion (OIF) are approximately equivalent. However, during the operating region, where a faulty measurement is encountered, both the estimated trajectories obtained based on CF and OIF significantly deviated from the ground truth. The variation of the MAV’s orientation in Euler angle representation is shown in Figure 7. The estimated orientation, obtained from the FR-OIF, is approximately close to the Vicon based ground truth. However, during the experiment, when the aerial robot was roaming around the rectangular path of trajectory, it was manoeuvring with small-angle variation along with the roll, pitch and yaw. Hence, the noticeable performance improvement for FR-OIF is prominent in transnational motion compared to that of the rotations.

In order to demonstrate the effectiveness of the proposed fault resilient framework, momentary faults are synthetically introduced into the measured sensor data obtained during the experiment. The evaluation is carried out in multiple scenarios depicting presence of spurious measurement from multiple sensors, as follows:

- **Case-1: Temporal fault only in LIO** measurement in between (20–30) s, while measurement from all other sensors are unaltered.
- **Case-2: Temporal fault only in VIO** measurement in between (50–60) s, while measurement from all other sensors are unaltered.
- **Case-3: Temporal fault in both LIO and UWB** measurements are itroduced in different operating points. The UWB measurement is faulty during (20–30) s, whereas for LIO reports faulty data during (35–45) s. Hence, this case evaluates multiple faults from different sensors in separate operating point.
- **Case-4: Simultaneous temporal failure of LIO and UWB** appeared during (20–30) s.

**Figure 8:**
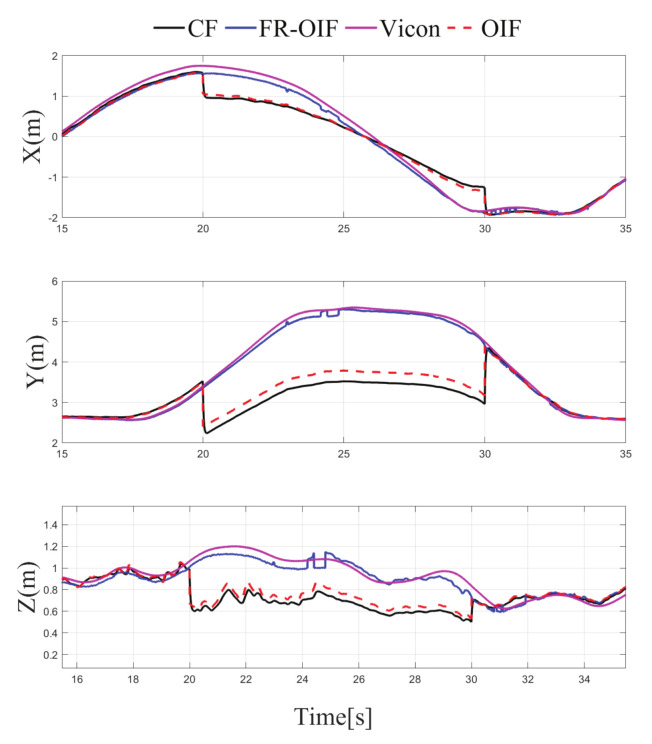
Case-1: Comparison of estimated position obtained from CF, OIF, FR-OIF and Vicon based ground truth, visualization in an emphasized mode of Figure 5 is presented. In the presence of a fault in the LIO for the duration of operation between (20–30) s, the centralized and classical OIF approach is unable to recover the failure in the estimated states, while the proposed FR-OIF successfully recovered from the faulty measurements and it is able to provide a close approximation of position estimate comparable with ground truth.

**Figure 9:**
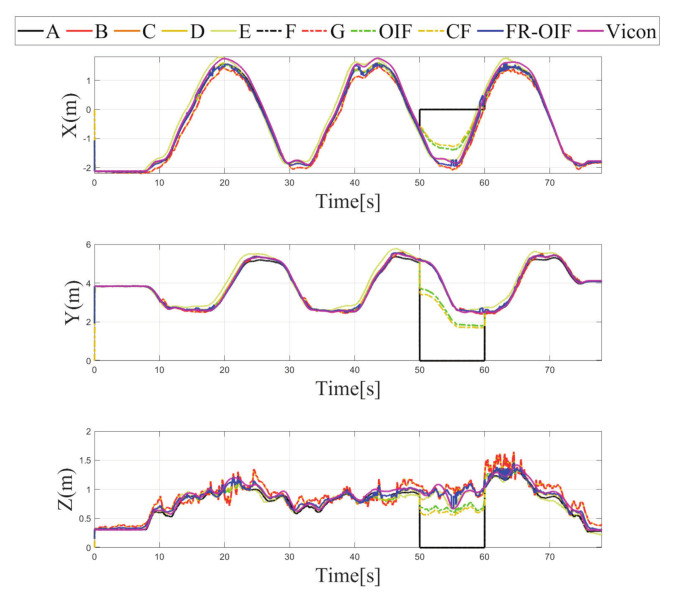
Case-2: Estimated positions along ‘X-Y-Z’ components obtained from intermediate nodes (A,⋯,G) and FR-OIF, compared with CF, OIF and the ground truth. The evaluation is carried out in presence of temporal fault appearing from VIO. Except the fused position obtained from FR-OIF all the estimated positions deviated during 50–60 s.

**Figure 10:**
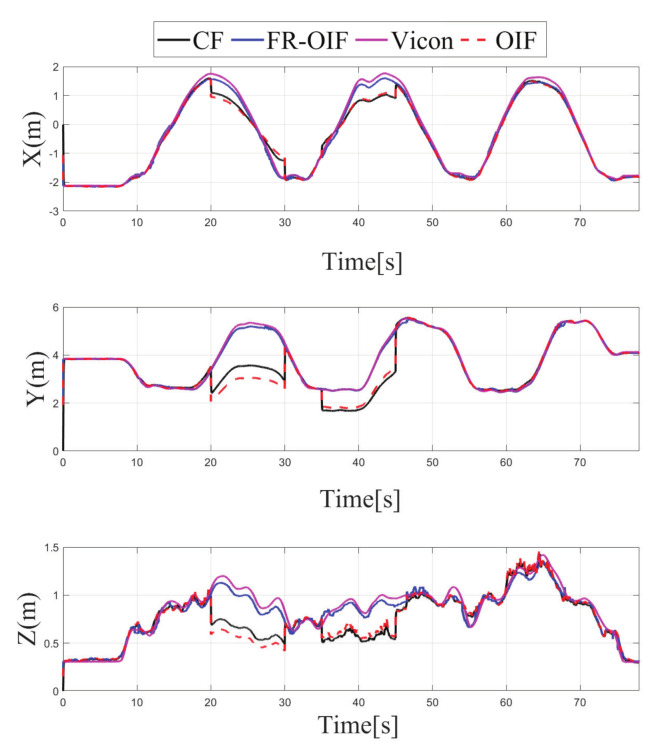
Case-3: Estimated positions along ‘X-Y-Z’ components obtained from intermediate nodes (A,⋯,G) and FR-OIF, compared with CF, OIF and the ground truth. The evaluation is carried out in presence of temporal fault appearing from UWB (20–30) s and LIO (35–45) s. Except the fused position obtained from FR-OIF all the estimated positions deviated in presence of faulty measurements.

**Figure 11:**
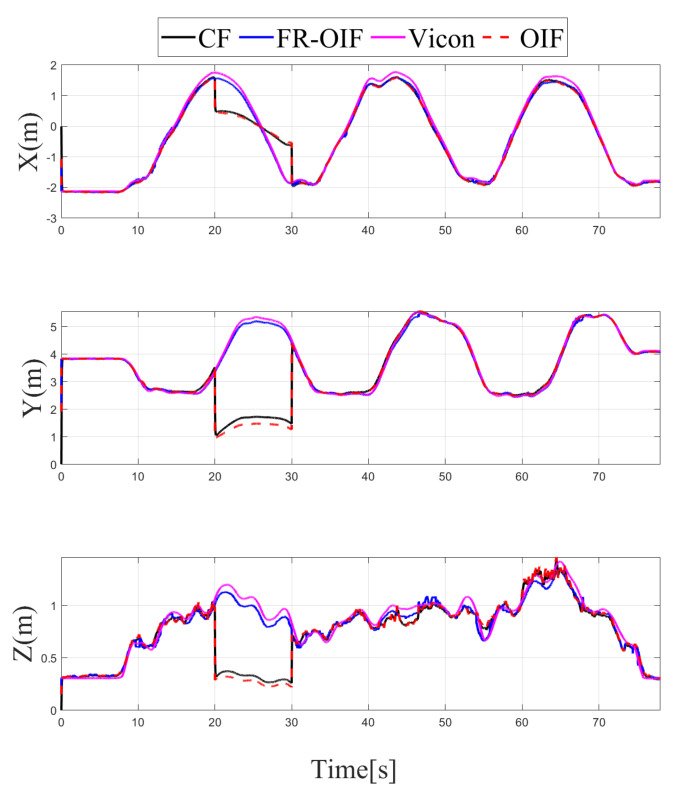
Case-4: Estimated positions along ‘X-Y-Z’ components obtained from intermediate nodes (A,⋯,G) and FR-OIF, compared with CF, OIF and the ground truth. The evaluation is carried out in presence of simultaneous temporal fault appearing from UWB and LIO during (20–30) s. Except the fused position obtained from FR-OIF all the estimated positions deviated in presence of faulty measurements.

Note that the sensor selected for reporting faulty operation and the corresponding time duration are arbitrarily selected without loss of generality. The variation of the estimated positions for all the possible cases under consideration is depicted in Figure 8, Figure 9, Figure 10 and Figure 11. It is evident from the results that the proposed FR-OIF is successfully capable of determining the MAV position in the presence of various possible failure conditions and temporal faulty sensor measurements. More significantly, from Figure 11 one can visualize that the FR-OIF demonstrated its efficacy for a simultaneous failure of multiple sensors at a time, as described in case-4.

The variation of estimated position, obtained from the different nodes A⋯G are also depicted in Figure 8, Figure 9, Figure 10 and Figure 11, corresponding to the various cases under consideration. Each of the node, individually employing an EKF, indeed eventually removes the measurement noise from the estimated pose. However, the accuracy of the corresponding node depends on the measurement of sensor associated with it. For example, if we consider the situation as described in the Case-1, the faulty measurement is associated with the LIO. Hence, the estimated position from the node *B* and *E*, which are using the position information based on LIO, (refer to Table 1) are inaccurate, as shown in Figure 6. Similarly, one can observe here that the estimated orientation, obtained from the nodes *D* and *F*, are erroneous during (20–30) s the period of the faulty operation of LIO, since these nodes are using the LIO based orientation as a measurements. The equivalent analysis also holds for the remaining case studies.

### 4.1. Comparison of FR-OIF with Centralized and Distributed Fusion Approaches

In order to demonstrate the efficiency of the proposed FR-OIF, comparative study with the EKF based centralized multi-sensor fusion as well as decentralized optimal information fusion is presented here. For the sake of completeness, a brief description of the EKF based centralized fusion architecture is described in the sequel. The centralized EKF follows exactly the same steps of the prediction and correction approach, as described in Equations (Section 3.1)–(Section 3.1), with the only exception that the measured pose from the multiple sensors are collectively taken into account. Hence, the output equation is described as:

(26) $$y_{k}\in R^{21\times 1}={\left[p_{{}_{LIO}},q_{{}_{LIO}},p_{{}_{VIO}},q_{{}_{VIO}},p_{{}_{UWB}},q_{{}_{IMU}}\right]}^{T}$$

The dimension of the measurement noise co-variance matrix $R_{k}$, Kalman gain $K_{k}$ and output gradient $H_{k}$ are redefined accordingly.

Apart from the EKF based centralized approach, a comparison study has been carried out with the existing decentralized OIF [31] method. The formulation presented in [31] considers a weighted sum of the individual node in the second layer fusion, as described in Equation (18). Both the CF and OIF are evaluated for all the scenarios presented in the Case 1–4 with faulty sensor measurements from multiple sensors. The variations of the estimated pose for CF, OIF and FR-OIF are presented in Figure 5, Figure 6, Figure 7 and Figure 8. The comparison study reveals that in the absence of sporadic measurements, the performance of all the methods (CF, OIF and FR-OIF) under consideration are approximately equivalent. However, in the presence of faulty measurements (Case 1–4), it can be observed that the estimated trajectory, obtained from the CF and OIF approach, deviates from the ground truth. Moreover, the presented experimental study brings out another interesting fact where the performance of the OIF closely resembles with the CF. This is highlighted in Figure 8, which is an emphasized version of Figure 6 for a time duration of operation under the faulty measurements (and the fact is also evident in other figures as well). In contrast, FR-OIF is capable of providing an accurate pose estimation even in the presence of fault from multiple sensors.

### 4.2. Accuracy in Terms of the Root Mean Square Error

**Figure 12:**
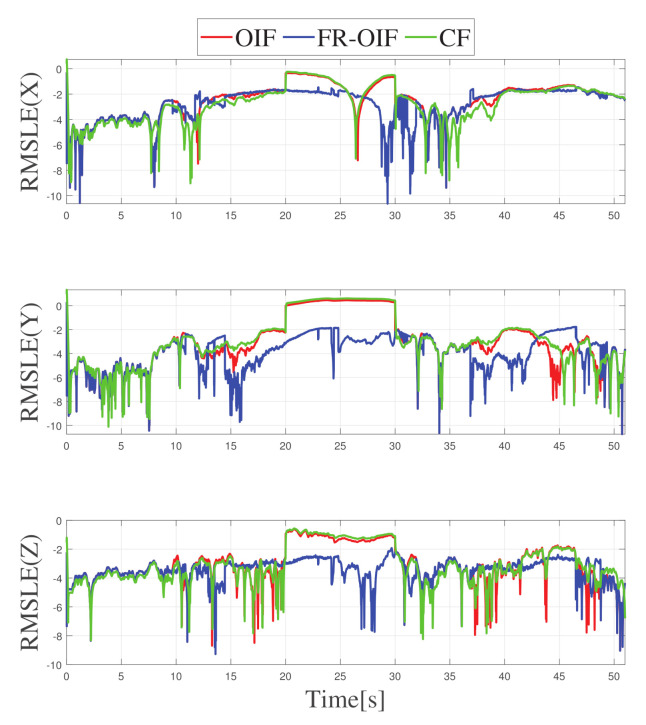
Variation of Semi-logarithmic root mean square error for position along X-Y-Z obtained from different fusion (CF, OIF, FR-OIF).

**Figure 13:**
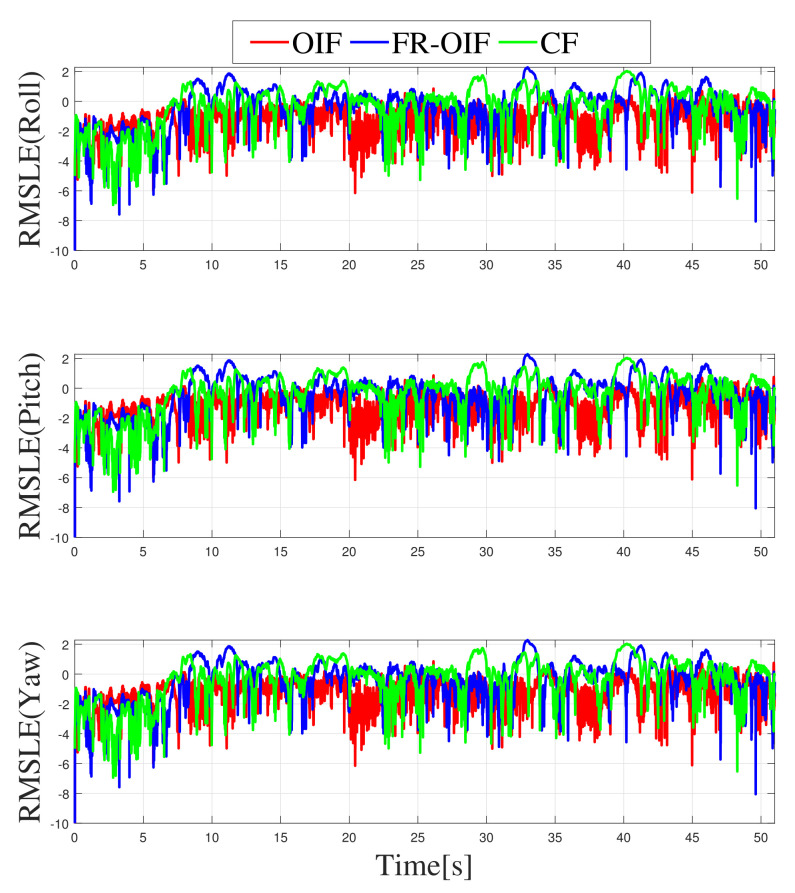
Semi-logarithmic root mean square error for orientation represented in Euler angles, obtained from different fusion (CF, OIF, FR-OIF).

In this section, the accuracy of the fusion algorithms are evaluated in terms of Root Mean Square Error (RMSE). In this case, a comparison is made in two steps. Firstly, the estimated poses are compared using a sliding window RMSE where the corresponding plots are depicted in Figure 12 and Figure 13 in a logarithmic scale. Secondly, to compare the performance of the estimated pose, obtained based on various nodes and fusion approaches, the single value RMSE is computed and illustrated in Table 2 and Table 3.

Note that the RMSE errors are computed by considering the Vicon based ground truth as reference. The RMSE comparison table proves that the second layered FR-OIF method is superior when the sensor measurements are erroneous. In order to visualize the variation of RMSE error along the trajectory, a sliding-window logarithmic RMSE (RMSLE) is considered with a window size of 100 samples. Since, the RMSLE provides the error in a logarithmic scale, the smaller the magnitude is (more negative) the more signifies for a higher accuracy. From the variation of RMSLE, as presented in Figure 12 and Figure 13. Even though the root mean square error for orientations has slightly varied with the efficiency of the estimators.

Hence, transnational motion during the experiment was made significant changes in the RMSE Table 2, as well as Figure 12, however it is not significantly visible in Table 3 and Figure 13.

The proposed FR-OIF provides excellent performance in terms of RMSE, compared to the centralized fusion approach. Moreover, based on the experimental results it can concluded that the proposed multi-sensor fusion is capable to provide a resilient pose estimation in the presence of faulty measurements and it has a great potential for various practical applications, involving multiple sensors and with a sufficient redundancy.

In the present context, the evaluation of the proposed fault resilient fusion is carried out with the experimental data, where temporal sensor fault are synthetically injected for the purpose of validation. However, part of the future work will consider evaluating the proposed FR-OIF sensor fusion framework in a field robotic experiment, where a momentary sensor failure is unavoidable in presence of dusty/smokey and dark environment. Additionally it is to be noted that, In the presented approach, and as in the case of most of the fault detection approaches in the related literature, the Δ has been ad hoc selected to a constant number and without loosing generality, while part of future work is also related to the adaptive determination of this value.

## 5. Conclusions

In this article, a novel decentralized multi-sensor fusion framework for resilient pose estimation of MAV is presented. The proposed multi-sensor fusion considered a two layered architecture. In the first layer, by combining the information from different sensors and by using an EKF a set of nodes are constructed. Each node provides an estimate of the MAV pose, which are collectively integrated by using OIF to provide an optimal estimate of it. Moreover, an unique fault isolation is embedded with the classical OIF formulation to incorporate the resiliency in presence of faulty measurements. Based on the experimental study an interesting fact has been established that, without an external fault isolation mechanism, the performance of the classical OIF closely resembles with the centralized EKF based multi-sensor fusion approach. Hence, these two methods are not sufficient to eliminate the fault accurately. In contrast, the proposed fault resilient optimal isolation technique is adequately capable to overcome such shortcomings. Even though the proposed FR-OIF is presented in this article is considered pose estimation of MAV, the formulation is quite generic and it can be applied in various autonomous navigation and with different robotic platforms and involving multiple sensors. 

## Figures and Tables

**Table 1:** Combination of sensor information used to construct the nodes of the proposed first layer

Pose	*A*	*B*	*C*	*D*	*E*	*F*	*G*
**Position**	Real sense Camera	3D LiDar	UWB	UWB	3D LiDar	Real sense Camera	UWB
**Orientation**	IMU	Real sense Camera	IMU	3D LiDar	IMU	3D- LiDar	Real sense Camera

**Table 2:** RMSE comparison for the estimation of the positions in meters.

Axis	*A*	*B*	*C*	*D*	*E*	*F*	*G*	CF	OIF	FR-OIF
X	0.1114	0.5740	0.2509	0.2509	0.5740	0.1114	0.2509	0.2503	0.2340	0.1394
Y	0.0806	2.1856	0.0793	0.0793	2.1856	0.0806	0.0793	0.7564	0.6452	0.0650
Z	0.0502	0.4616	0.1122	0.1122	0.4616	0.0502	0.1122	0.1751	0.1557	0.0499

**Table 3:** RMSE comparison for the estimation of the orientation in Euler angles.

Angles	*A*	*B*	*C*	*D*	*E*	*F*	*G*	CF	OIF	FR-OIF
ψ	0.4667	0.7522	0.4667	0.7473	0.4667	0.7473	0.0168	0.5343	0.5154	0.5340
θ	2.0663	2.1664	2.0663	1.9324	2.0663	1.9324	0.0188	1.9618	1.9618	1.9505
ϕ	1.9387	1.9246	1.9387	1.9357	1.9387	1.9357	0.0066	1.8365	1.8456	1.8341

## Data Availability

Not applicable.

## References

[1] Wanasinghe T.R., Mann G.K., Gosine R.G. Decentralized cooperative localization for heterogeneous multi-robot system using split covariance intersection filter. Proceedings of the 2014 Canadian Conference on Computer and Robot Vision.

[2] Durrant-Whyte H.F., Rao B., Hu H. Toward a fully decentralized architecture for multi-sensor data fusion. Proceedings of the IEEE International Conference on Robotics and Automation.

[3] Rigatos G.G. (2010). Extended Kalman and particle filtering for sensor fusion in motion control of mobile robots. Math. Comput. Simul..

[4] Yazdkhasti S., Sasiadek J. (2017). Multi Sensor Fusion Based on Adaptive Kalman Filtering.

[5] Brena R.F., Aguileta A.A., Trejo L.A., Molino-Minero-Re E., Mayora O. (2020). Choosing the Best Sensor Fusion Method: A Machine-Learning Approach. Sensors.

[6] Liggins M., Hall D., Llinas J. (2017). Handbook of Multisensor Data Fusion: Theory and Practice.

[7] Hall D., Chong C.Y., Llinas J., Liggins M. (2017). Distributed Data Fusion for Network-Centric Operations.

[8] Hoang T., Duong P., Van N., Viet D., Vinh T. Multi-sensor perceptual system for mobile robot and sensor fusion-based localization. Proceedings of the 2012 International Conference on Control, Automation and Information Sciences (ICCAIS).

[9] Vasquez B.P.E.A., Gonzalez R., Matia F., De la Puente P. (2018). Sensor fusion for tour-guide robot localization. IEEE Access.

[10] Mueller M.W., Hamer M., D’Andrea R. Fusing ultra-wideband range measurements with accelerometers and rate gyroscopes for quadrocopter state estimation. Proceedings of the 2015 IEEE International Conference on Robotics and Automation (ICRA).

[11] Al Khatib E.I., Jaradat M.A., Abdel-Hafez M., Roigari M. Multiple sensor fusion for mobile robot localization and navigation using the Extended Kalman Filter. Proceedings of the 2015 10th International Symposium on Mechatronics and Its Applications (ISMA).

[12] Cotugno G., D’Alfonso L., Lucia W., Muraca P., Pugliese P. Extended and Unscented Kalman Filters for mobile robot localization and environment reconstruction. Proceedings of the 21st Mediterranean Conference on Control and Automation.

[13] Anjum M.L., Park J., Hwang W., Kwon H.i., Kim J.H., Lee C., Kim K.S. Sensor data fusion using unscented kalman filter for accurate localization of mobile robots. Proceedings of the ICCAS 2010.

[14] Ullah I., Shen Y., Su X., Esposito C., Choi C. (2019). A localization based on unscented Kalman filter and particle filter localization algorithms. IEEE Access.

[15] D’Alfonso L., Lucia W., Muraca P., Pugliese P. (2015). Mobile robot localization via EKF and UKF: A comparison based on real data. Robot. Auton. Syst..

[16] Martinelli F. Robot localization: Comparable performance of EKF and UKF in some interesting indoor settings. Proceedings of the 2008 16th Mediterranean Conference on Control and Automation.

[17] Wang S., Chen L., Gu D., Hu H. (2014). An optimization based moving horizon estimation with application to localization of autonomous underwater vehicles. Robot. Auton. Syst..

[18] Kimura K., Hiromachi Y., Nonaka K., Sekiguchi K. Vehicle localization by sensor fusion of LRS measurement and odometry information based on moving horizon estimation. Proceedings of the 2014 IEEE Conference on Control Applications (CCA), Juan Les Antibes.

[19] Zhou B., Qian K., Fang F., Ma X., Dai X. Multi-sensor fusion robust localization for indoor mobile robots based on a set-membership estimator. Proceedings of the 2015 IEEE International Conference on Cyber Technology in Automation, Control and Intelligent Systems (CYBER).

[20] Fang X., Wang C., Nguyen T.M., Xie L. (2020). Graph optimization approach to range-based localization. IEEE Trans. Syst. Man Cybern. Syst..

[21] Nguyen T.M., Cao M., Yuan S., Lyu Y., Nguyen T.H., Xie L. (2021). Viral-fusion: A visual-inertial-ranging-lidar sensor fusion approach. IEEE Trans. Robot..

[22] Nebot E.M., Bozorg M., Durrant-Whyte H.F. (1999). Decentralized architecture for asynchronous sensors. Auton. Robot..

[23] Alatise M.B., Hancke G.P. (2020). A review on challenges of autonomous mobile robot and sensor fusion methods. IEEE Access.

[24] Zali A., Bozorg M., Masouleh M.T. Localization of an indoor mobile robot using decentralized data fusion. Proceedings of the 2019 7th International Conference on Robotics and Mechatronics (ICRoM).

[25] Santos M.C., Santana L.V., Martins M.M., Brandão A.S., Sarcinelli-Filho M. Estimating and controlling uav position using rgb-d/imu data fusion with decentralized information/kalman filter. Proceedings of the 2015 IEEE International Conference on Industrial Technology (ICIT).

[26] Li H., Nashashibi F. (2013). Cooperative multi-vehicle localization using split covariance intersection filter. IEEE Intell. Transp. Syst. Mag..

[27] Sijs J., Lazar M., Bosch P. State fusion with unknown correlation: Ellipsoidal intersection. Proceedings of the 2010 American Control Conference.

[28] Wu M., Ma H., Zhang X. (2018). Decentralized cooperative localization with fault detection and isolation in robot teams. Sensors.

[29] Carrillo-Arce L.C., Nerurkar E.D., Gordillo J.L., Roumeliotis S.I. Decentralized multi-robot cooperative localization using covariance intersection. Proceedings of the 2013 IEEE/RSJ International Conference on Intelligent Robots and Systems.

[30] Wang X., Sun S., Li T., Liu Y. (2021). Fault tolerant multi-robot cooperative localization based on covariance union. IEEE Robot. Autom. Lett..

[31] Sun S.L., Deng Z.L. (2004). Multi-sensor optimal information fusion Kalman filter. Automatica.

[32] Bakr M.A., Lee S. (2017). Distributed multisensor data fusion under unknown correlation and data inconsistency. Sensors.

[33] Al Hage J., El Najjar M.E., Pomorski D. (2017). Multi-sensor fusion approach with fault detection and exclusion based on the Kullback–Leibler Divergence: Application on collaborative multi-robot system. Inf. Fusion.

[34] Li T., Corchado J.M., Sun S. (2018). Partial consensus and conservative fusion of Gaussian mixtures for distributed PHD fusion. IEEE Trans. Aerosp. Electron. Syst..

[35] Rekleitis I. (2003). Cooperative Localization and Multi-Robot Exploration. Ph.D. Thesis.

[36] Kshirsagar J., Shue S., Conrad J.M. A survey of implementation of multi-robot simultaneous localization and mapping. Proceedings of the SoutheastCon 2018.

[37] Perron J.M., Huang R., Thomas J., Zhang L., Tan P., Vaughan R.T. Orbiting a moving target with multi-robot collaborative visual slam. Proceedings of the Workshop on Multi-View Geometry in Robotics (MVIGRO).

[38] Shan T., Englot B., Meyers D., Wang W., Ratti C., Daniela R. LIO-SAM: Tightly-coupled Lidar Inertial Odometry via Smoothing and Mapping. Proceedings of the IEEE/RSJ International Conference on Intelligent Robots and Systems (IROS).

[39] Corke P.I., Khatib O. (2011). Robotics, Vision and Control: Fundamental Algorithms in MATLAB.

[40] Sola J. (2017). Quaternion kinematics for the error-state Kalman filter. arXiv.

[41] Simon D. (2006). Optimal State Estimation: Kalman, H Infinity, and Nonlinear Approaches.

[42] Givens M.W., Coopmans C. A survey of inertial sensor fusion: Applications in suas navigation and data collection. Proceedings of the 2019 International Conference on Unmanned Aircraft Systems (ICUAS).

[43] Yuksel G., Isik O.R. (2015). Numerical analysis of Backward–Euler discretization for simplified magnetohydrodynamic flows. Appl. Math. Model..

[44] Rao S.S. (2019). Engineering Optimization: Theory and Practice.

